# Physical chronic conditions, multimorbidity and sedentary behavior amongst middle-aged and older adults in six low- and middle-income countries

**DOI:** 10.1186/s12966-017-0602-z

**Published:** 2017-10-27

**Authors:** Davy Vancampfort, Brendon Stubbs, Ai Koyanagi

**Affiliations:** 1 KU Leuven Department of Rehabilitation Sciences, Tervuursevest 101, 3001 Leuven, Belgium; 2 KU Leuven, University Psychiatric Center KU Leuven, Leuvensesteenweg 517, 3070 Kortenberg, Belgium; 3 Physiotherapy Department, South London and Maudsley NHS Foundation Trust, Denmark Hill, London, SE5 8AZ UK; 4 Health Service and Population Research Department, Institute of Psychiatry, Psychology and Neuroscience, King’s College London, De Crespigny Park, London, Box SE5 8AF UK; 5 Faculty of Health, Social Care and Education, Anglia Ruskin University, Chelmsford, UK; 6 Research and Development Unit, Parc Sanitari Sant Joan de Déu, Universitat de Barcelona, Fundació Sant Joan de Déu, Dr. Antoni Pujadas, 42, Sant Boi de Llobregat, 0883 Barcelona, Spain; 7 Instituto de Salud Carlos III, Centro de Investigación Biomédica en Red de Salud Mental, CIBERSAM, Monforte de Lemos 3-5 Pabellón 11, 28029 Madrid, Spain

**Keywords:** Multimorbidity, Pain, Mobility limitation, Sitting, Sedentary time

## Abstract

**Background:**

Sedentary behavior (SB) is, irrespective of a person’s physical activity levels, associated with a wide range of deleterious outcomes such as diabetes, stroke and associated premature mortality. There are no nationally representative, multi-national, population-based studies investigating the relationship between SB, chronic conditions, and physical multimorbidity (i.e., two or more chronic physical conditions). Thus, this cross-sectional study aimed to assess the association between chronic conditions, physical multimorbidity and SB among community-dwelling adults in six low- and middle-income countries (LMICs). We also explored the influential factors of these relationships.

**Method:**

The Study on Global Ageing and Adult Health (SAGE) survey included 34,129 adults aged ≥50 years. SB was self-reported and expressed as a categorical variable [<8 or ≥8 h per day (high SB)]. Eleven chronic physical conditions (angina, arthritis, asthma, chronic back pain, chronic lung disease, diabetes, edentulism, hearing problems, hypertension, stroke, visual impairment) were assessed. Multivariable logistic regression and mediation analyses were conducted.

**Results:**

The prevalence of physical multimorbidity and high SB (≥8 h/day) were 45.5% (43.7%–47.4%) and 10.8% (9.7%–12.1%), respectively. The prevalence of high SB increased in a linear fashion from 7.1% in people with no chronic condition to 24.1% in those with ≥4 chronic conditions. In the multivariable analysis, visual impairment (OR = 2.62), stroke (OR = 2.02), chronic back pain (OR = 1.70) hearing problems (OR = 1.58), chronic lung disease (OR = 1.48), asthma (OR = 1.39), arthritis (OR = 1.22) and multimorbidity (OR = 1.41) were significantly associated with high SB. Disability explained more than 50% of the association for all chronic conditions with particularly high percentages (>80%) for arthritis, asthma, and multimorbdity. Mobility problems explained 88.1% and 85.1% of the association of SB with arthritis and physical multimorbidiy, respectively. Pain was highly influential in the SB-arthritis relationship (85.6%). Sleep/energy problems explained between 9.3% (stroke) to 49.1% (arthritis) of the association, and cognitive problems from 21.5% (stroke) to 33.4% (hearing problems). Findings for anxiety and depression were mixed.

**Conclusion:**

In LMICs, those with chronic conditions and physical multimorbidity are significantly more sedentary. Targeted messages to reduce time spent sedentary among individuals with chronic conditions may ameliorate associated disability, mobility difficulties and pain that are themselves the most important risk factors for SB.

**Electronic supplementary material:**

The online version of this article (10.1186/s12966-017-0602-z) contains supplementary material, which is available to authorized users.

## Background

The average life expectancy is increasing worldwide [1]. Irrespective of the socioeconomic development of a country, the main causes of death and disability in middle-aged to older adults (i.e. 50 years or older) are non-communicable diseases (NCDs), many of which often occur in combination [2]. The co-occurrence of two or more chronic physical conditions is known as physical multimorbidity [3]. With increasing population and life expectancy, the disease burden of physical multimorbidity to both individuals and societies are increasing [4]. Although data are scarce, in low- and middle-income countries (LMICs) about half of middle-aged to older adults experience physical multimorbidity (i.e. 2 or more chronic conditions), about 25% have at least three, and about 10% four or more chronic conditions [5]. Physical multimorbidity is an important risk concept as it is associated with a lower quality of life [6], increased health-care utilization and costs [7], and ultimately, higher risk for premature mortality [8]. There is a consensus that in the years to come, this disease burden will be greatest in LMICs [9], but knowledge on physical multimorbidity from LMICs is limited compared with HICs (95% of the available studies are from HICs), despite the fact that nearly 80% of NCD related deaths occur in LMICs [10].

The promotion of physical activity is an essential strategy within the multifaceted care of middle-aged to older adults for the prevention and management of chronic disease and physical multimorbidity [11]. Regular physical activity contributes to the primary and secondary prevention of a wide range of chronic diseases [12], improves quality of life [13], and is associated with reduced risk of premature death [14]. There is also increasing evidence demonstrating that sedentary behavior (SB) should be considered in this multifaceted care as it is, irrespective of a person’s physical activity levels, associated with a wide range of deleterious outcomes such as diabetes, stroke and associated premature mortality [15]. SB refers to any waking behavior characterized by an energy expenditure ≤1.5 metabolic equivalents (METs), while in a sitting, reclining or lying posture [16]. SB is highly prevalent and pervasive in societies across the world [17, 18]. A recent systematic review, virtually exclusively among western societies, demonstrated that middle-aged to older adults spend 65–80% of their waking day being sedentary [19].

Research investigating the relationship between SB and chronic diseases has solely focused on high-income countries. In addition, there is a particular dearth of research on SB and physical multimorbidity. In the only study on this topic to date, among 2048 American adults (mean age = 42.7 years; 50.9% male), every 60 min/day increase in SB was associated with an 11% (adjusted OR =1.11; 95%CI = 1.01–1.21; *P* = 0.03) increased odds of being multimorbid (i.e. having ≥2 morbidities) [20]. Moreover, the authors found that SB was associated with multimorbidity independent of light-intensity physical activity and adherence to moderate-to-vigorous physical activity guidelines, which underscores the importance of minimizing prolonged SB (in addition to promoting physical activity) in the treatment of multimorbidity.

The lack of nationally representative population-based studies investigating the associations between SB, chronic conditions, and physical multimorbidity in LMICs is an important research gap given the rapid increase in chronic diseases in these countries, mainly due to changes in lifestyle [21]. Furthermore, the association between chronic conditions or multimorbidity on SB may differ in LMICs due to different disease profiles [22], suboptimal treatment of chronic conditions [23, 24], and differences in knowledge regarding the risks of being sedentary [25]. In addition, at the population level, there is a paucity of information on factors that might influence the relationship between SB, chronic diseases and physical multimorbidity. In this study we focused on physical (mobility, disability, pain and discomfort) and mental (cognition, sleep and energy, anxiety and depression) conditions as these variables have all been associated with SB [26, 27] and the presence of chronic conditions [5]. Given the aforementioned gaps within the literature, we aimed to assess the association between chronic conditions or physical multimorbidity and SB among community-dwelling adults aged 50 or older in six LMICs, and to assess the factors that might influence this relationship. We hypothesize that higher levels of SB are associated with the presence of chronic conditions and physical multimorbidity.

## Methods

### The survey

Data from the SAGE were analyzed. This survey was undertaken in China, Ghana, India, Mexico, Russia, and South Africa between 2007 and 2010. Based on the World Bank classification at the time of the survey, all of these countries were LMICs. Details of the survey methodology have been published elsewhere [28]. In brief, in order to obtain nationally representative samples, a multistage clustered sampling design method was used. The sample consisted of adults aged ≥18 years with oversampling of those aged ≥50 years. Trained interviewers conducted face-to-face interviews using a standard questionnaire. Standard translation procedures were undertaken to ensure comparability between countries. If a respondent was unable to undertake the interview because of limited cognitive function, then a separate questionnaire was administered to a proxy respondent. These individuals were not included in the current study. The survey response rate ranged from 51% (Mexico) to 93% (China). Sampling weights were constructed to adjust for the population structure as reported by the United Nations Statistical Division. Ethical approval was obtained from the WHO Ethical Review Committee and local ethics research review boards. Written informed consent was obtained from all participants.

### Sedentary behaviour (outcome variable)

In order to assess SB, participants were asked to state the total time they usually spent (expressed in minutes per day) sitting or reclining including at work, at home, getting to and from places, or with friends (e.g. sitting at a desk, sitting with friends, travelling in car, bus, train, reading, playing cards or watching television). This did not include time spent sleeping. The variable on SB was used in the analysis as a categorical [<8 (coded 0) or ≥8 h (coded 1) per day] variable. The eight-hour cut-off was chosen as previous research indicated that being sedentary for ≥8 h/day in the general population is associated with a higher risk for premature mortality [29].

### Chronic conditions and physical multimorbidity (exposures)

Eleven chronic physical conditions (angina, arthritis, asthma, chronic back pain, chronic lung disease, diabetes, edentulism, hearing problems, hypertension, stroke, visual impairment) were assessed. Chronic back pain was defined as having had back pain everyday during the last 30 days. Respondents who answered affirmatively to the question “Have you lost all of your natural teeth?” were considered to have edentulism. The participant was considered to have hearing problems if the interviewer observed this condition during the survey. Blood pressure was measured three times with a one-minute interval with the use of a wrist blood pressure monitor (Medistar Wrist Blood Pressure Model S) and the mean value of the three measurements was calculated. Hypertension was defined as having at least one of the following: systolic blood pressure ≥ 140 mmHg; diastolic blood pressure ≥ 90 mmHg; or self-reported diagnosis. Visual impairment was defined as having extreme difficulty in seeing and recognizing a person that the participant knows across the road [30]. A validity study showed that this response likely corresponds to WHO definitions of visual impairment (20/60 or 0.48 logMAR) [30]. Diabetes and stroke were solely based on lifetime self-reported diagnosis.

For other conditions, the participant was considered to have the condition in the presence of either one of the following: self-reported diagnosis; or symptom-based diagnosis based on algorithms. We used these algorithms, which have been used in previous studies using the same dataset, to detect undiagnosed cases [31, 32]. Specifically, the validated Rose questionnaire was used for angina [33], and other previously validated symptom-based algorithms were used for arthritis, asthma, and chronic lung disease [31, 34]. The questions used to assess self-reported diagnosis and the symptom-based algorithms are provided in Additional file 1: Tables S1 and S2. The total number of chronic conditions was calculated and categorized as 0, 1, 2, 3, and ≥4. Multimorbidity was defined as ≥2 chronic conditions [32].

### Mediators

#### Health status

Health status was evaluated with eight health-related questions pertaining to four health domains including: (a) mobility; (b) pain and discomfort; (c) cognition; and (d) sleep and energy. Each of the four domains corresponds to those in common health related quality of life outcome measures such as the Short Form-12 (SF-12) [35], the Health Utilities Index Mark-3 (HUI) [36] and the EUROQOL-5D [37]. Each domain consists of two questions that assessed health function in the past 30 days. The actual questions can be found in Additional file 1: Table S3. Each item was scored on a five-point scale ranging from ‘none’ to ‘extreme/cannot do’. For each separate domain, we used factor analysis with polychoric correlations to obtain a factor score which was later converted to scores ranging from 0 to 100 with higher values representing worse health function [38, 39].

#### Anxiety

In accordance with previous publications using a dataset with the identical question, those who claimed to have severe/extreme problems with worry or anxiety in the past 30 days were considered to have anxiety [40, 41].

#### Depression

Questions based on the World Mental Health Survey version of the Composite International Diagnostic Interview [42] were used for the endorsement of past 12-month DSM-IV depression using the same algorithm used in previous studies using the same dataset [32, 43] (Details provided in Additional file 1: Table S4).

#### Disability

Disability was assessed by the use of the 12-item validated version of the World Health Organization Disability Assessment Schedule 2.0 (WHODAS 2.0) [44]. Item Response Theory analysis was used to create a scale ranging from 0 (no disability) to 100 (maximum disability) [45].

### Control variables

These included sex, age, wealth, highest level of education achieved (≤primary, secondary, ≥tertiary), setting (urban or rural), living arrangement (alone or not), and employment status (engaged in paid work ≥2 days in last 7 days: Y/N). Wealth quintiles were created based on country-specific income.

### Statistical analysis

The statistical analysis was performed with Stata 14.1 (Stata Corp LP, College station, Texas). The analysis was restricted to those aged ≥50 years given the high prevalence of chronic physical conditions in this age group. We conducted multivariable logistic regression analysis to assess the association between the number of chronic conditions including physical multimorbidity (≥2 chronic physical conditions) or each of the 11 chronic conditions (exposure variables) and sedentary behavior (outcome). Analyses using the overall sample and by age groups (50–64, ≥65 years) was done. Next, in order to gain an understanding of the extent to which various factors may explain the relation of individual chronic conditions and physical multimorbidity with SB, we conducted mediation analysis using the overall sample. We did not conduct this analysis for angina, diabetes, edentulism, and hypertension as these conditions were not significantly associated with SB in the overall sample. We focused on anxiety, cognition, depression, disability, mobility, pain/discomfort, and sleep/energy for their previously reported association with the exposure (chronic physical conditions) and the outcome (sedentary behavior). We used the *khb* (Karlson Holm Breen) command in Stata [46] for this purpose. This method can be applied in logistic regression models and decomposes the total effect (i.e., unadjusted for the influential factor) of a variable into direct (i.e. the effect of chronic conditions or multimorbidity on SB adjusted for the factor) and indirect effects (i.e. the mediational effect). Using this method, the percentage of the main association explained by the mediator can also be calculated. Each potential mediator was included in the model individually. All regression analyses were adjusted for sex, age, education, wealth, setting, unemployment, living arrangement, and country. Country adjustment was done by including dummy variables for each country. When the individual chronic conditions were the exposure variable, the models were also adjusted for the presence of other chronic illness to account for comorbid chronic conditions. This variable included information on whether the individual had any of the other ten chronic conditions (Y/N). All variables were included in the models as categorical variables with the exception of age, pain/discomfort, cognition, sleep/energy, and disability (continuous variables). Under 5% of the data were missing for the variables used in the analysis. Complete-case analysis was done. The sample weighting and the complex study design were taken into account in the analyses. Results from the regression analyses are presented as odds ratios (ORs) with 95% confidence intervals (CIs). The level of statistical significance was set at *P* < 0.05.

## Results

The final analytical sample consisted of 34,129 individuals (China *n* = 13,175; Ghana *n* = 4305; India *n* = 6560; Mexico *n* = 2313; Russia *n* = 3938; South Africa *n* = 3838) aged ≥50 years. The median (IQR) age was 62 (55–70) years and 47.9% were males. The prevalence of physical multimorbidity and high SB (i.e. ≥8 h/day) were 45.5% (43.7%–47.4%) and 10.8% (9.7%–12.1%), respectively (Table 1).

**Table 1:** Sample characteristics (overall and by highly sedentary behavior)

				Highly sedentary behavior^a^	
Characteristic	Category	N	Overall	No	Yes
Number of chronic conditions	0	6624	22.2	23.2	14.6
	1	11,437	32.3	33.3	24.1
	2	7430	22.4	22.6	20.7
	3	3880	12.2	11.7	16.4
	≥4	3214	10.9	9.2	24.1
Sociodemographics					
Age (years)	Median (IQR)		62 (55–70)	61 (55–70)	67 (58–75)
Sex	Male	15,666	47.9	48.4	45.1
Education	≤Primary	21,264	57.4	58.8	47.7
	Secondary	9943	35.2	34.1	41.8
	≥Tertiary	2189	7.4	7.1	10.5
Wealth	Poorest	6496	17.1	16.6	20.7
	Poorer	6697	19.0	18.5	21.7
	Middle	6648	19.5	19.6	19.1
	Richer	7009	21.3	21.8	18.0
	Richest	7147	23.1	23.5	20.5
Setting	Urban	17,115	46.2	45.4	51.5
Unemployed	Yes	20,134	57.3	55.7	70.9
Living arrangement	Alone	3608	10.0	8.8	18.8
Chronic physical conditions					
Angina	Yes	4921	17.6	16.4	25.5
Arthritis	Yes	9514	29.5	28.5	37.3
Asthma	Yes	2390	7.9	7.5	10.7
Chronic back pain	Yes	2543	8.6	7.7	15.9
Chronic lung disease	Yes	4477	15.8	14.5	25.2
Diabetes	Yes	2647	6.8	6.7	8.4
Edentulism	Yes	4124	12.9	12.1	19.7
Hearing problems	Yes	2075	5.6	4.9	11.1
Hypertension	Yes	20,141	55.0	54.0	61.9
Stroke	Yes	1190	3.0	2.6	7.0
Visual impairment	Yes	353	1.3	1.0	3.3
Health status					
Mobility	Median (IQR)		27 (0–58)	27 (0–51)	51 (27–73)
Pain/discomfort	Median (IQR)		36 (0–56)	36 (0–46)	36 (18–56)
Cognition	Median (IQR)		50 (40–69)	31 (0–50)	41 (20–60)
Sleep/energy	Median (IQR)		20 (0–49)	20 (0–40)	40 (0–59)
Other variables					
Disability	Median (IQR)		11 (3–31)	11 (3–28)	25 (8–47)
Anxiety	Yes	2046	8.1	7.5	13.6
Depression	Yes	1692	6.0	5.8	8.7

*Abbreviation: IQR* Interquartile range

Data are column percentages unless otherwise stated

Estimates are based on weighted sample

^a^Highly sedentary behavior referred to being sedentary for ≥8 h/day

**Fig. 1:**
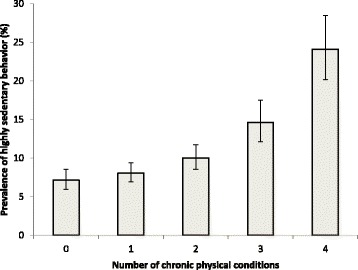
Prevalence of highly sedentary behavior by number of chronic physical conditions. Estimates are based on weighted sample. Bars denote 95% confidence intervals. Highly sedentary behavior referred to being sedentary for ≥8 hours/day.

The prevalence of high SB increased in a linear fashion with an increasing number of chronic conditions ranging from 7.1% in people with no chronic condition to 24.1% in those with 4 or more chronic conditions (Fig. 1).

The results of the multivariable logistic regression analysis assessing the association between chronic conditions or physical multimorbidity and high SB are presented in Table 2. In the overall sample, visual impairment (OR = 2.62), stroke (OR = 2.02), chronic back pain (OR = 1.70) hearing problems (OR = 1.58), chronic lung disease (OR = 1.48), asthma (OR = 1.39), arthritis (OR = 1.22), and physical multimorbidity (OR = 1.41) were significantly associated with high SB. When the analysis was stratified by age groups, arthritis, chronic back pain, hearing problems, and visual impairment, and physical multimorbidity were only significantly associated with high SB in the older age group, while asthma was only associated with high SB in the middle-aged group (*P* < 0.05). In terms of the number of chronic conditions, in the overall sample, compared to those who no chronic conditions, the presence of one or 2 chronic conditions was not significantly associated with high SB but the presence of 3 (OR = 1.55; *p* < 0.001) and 4 or more (OR = 2.22; p < 0.001) was associated with high SB. When stratified by age groups, in those aged 65 years or older, having 2 chronic conditions was significantly associated with higher odds (OR = 1.67) for high SB, while this odds increased to 2.36 and 2.98 for the presence of 3 and 4 or more chronic conditions, respectively (*P* < 0.001).

**Table 2:** Associations of chronic conditions, multimorbidity, and number of chronic conditions with highly sedentary behavior (outcome) estimated by multivariable logistic regression

		Overall	50–64 years	≥65 years
Angina	Yes vs. No	1.04 [0.83,1.31]	1.00 [0.71,1.41]	1.10 [0.84,1.45]
Arthritis	Yes vs. No	1.22* [1.03,1.44]	1.17 [0.92,1.49]	1.33* [1.07,1.67]
Asthma	Yes vs. No	1.39* [1.07,1.80]	1.59* [1.02,2.47]	1.24 [0.95,1.61]
Chronic back pain	Yes vs. No	1.70*** [1.37,2.11]	1.38 [0.98,1.95]	1.87*** [1.43,2.44]
Chronic lung disease	Yes vs. No	1.48*** [1.19,1.85]	1.69*** [1.26,2.26]	1.38* [1.05,1.82]
Diabetes	Yes vs. No	1.15 [0.93,1.44]	1.24 [0.89,1.72]	1.15 [0.88,1.49]
Edentulism	Yes vs. No	1.11 [0.86,1.43]	0.87 [0.61,1.26]	1.14 [0.84,1.54]
Hearing problems	Yes vs. No	1.58*** [1.23,2.02]	1.20 [0.68,2.11]	1.54** [1.16,2.03]
Hypertension	Yes vs. No	0.98 [0.85,1.14]	0.98 [0.79,1.22]	1.03 [0.85,1.26]
Stroke	Yes vs. No	2.02*** [1.53,2.67]	2.20*** [1.46,3.32]	2.09*** [1.46,3.00]
Visual impairment	Yes vs. No	2.62*** [1.75,3.93]	2.11 [0.86,5.19]	2.45*** [1.52,3.95]
Multimorbidity^a^	Yes vs. No	1.41*** [1.19,1.66]	1.17 [0.93,1.48]	1.81*** [1.39,2.36]
Number of chronic conditions	0	1.00	1.00	1.00
	1	1.09 [0.88,1.37]	1.07 [0.82,1.40]	1.35 [0.97,1.88]
	2	1.16 [0.93,1.46]	1.01 [0.75,1.36]	1.67* [1.07,2.61]
	3	1.55*** [1.21,1.98]	1.10 [0.69,1.74]	2.36*** [1.58,3.52]
	≥4	2.22*** [1.68,2.94]	2.26*** [1.44,3.53]	2.98*** [1.96,4.51]

Data are odds ratio [95% confidence interval]

Highly sedentary behavior referred to being sedentary for ≥8 h/day

Models are adjusted for sex, age, education, wealth, setting, unemployment, living arrangement, and country. For individual chronic conditions, the models were also adjusted for the presence of other chronic conditions

* *p* < 0.05, ** *p* < 0.01, *** *p* < 0.001

^a^Multimorbidity was defined as ≥2 chronic conditions

The results of the mediation analysis among those aged ≥50 years are shown in Tables 3 and 4.

**Table 3:** Mental health (anxiety, cognition, depression, sleep/energy) variables as mediators in the association between chronic conditions (multimorbidity) and highly sedentary behavior among adults aged ≥50 years

		Total effect		Direct effect		Indirect effect		
Exposure	Mediator	OR [95% CI]	*P*-value	OR [95% CI]	*P*-value	OR [95% CI]	*P*-value	% Mediated
Arthritis	Anxiety	1.22 [1.04,1.45]	0.017	1.21 [1.03,1.43]	0.023	1.01 [1.00,1.02]	0.064	NA
	Cognition	1.23 [1.04,1.46]	0.014	1.15 [0.97,1.37]	0.108	1.07 [1.04,1.10]	<0.001	32.8
	Depression	1.22 [1.04,1.45]	0.018	1.21 [1.02,1.43]	0.030	1.02 [1.01,1.03]	0.003	7.7
	Sleep/energy	1.23 [1.04,1.45]	0.017	1.11 [0.94,1.31]	0.225	1.11 [1.07,1.14]	<0.001	49.1
Asthma	Anxiety	1.38 [1.06,1.81]	0.017	1.30 [0.99,1.70]	0.059	1.06 [1.03,1.10]	<0.001	19.3
	Cognition	1.40 [1.07,1.83]	0.014	1.27 [0.97,1.66]	0.082	1.10 [1.06,1.14]	<0.001	28.7
	Depression	1.39 [1.06,1.81]	0.016	1.33 [1.02,1.74]	0.036	1.04 [1.02,1.07]	0.003	13.2
	Sleep/energy	1.38 [1.07,1.79]	0.014	1.21 [0.93,1.56]	0.155	1.15 [1.10,1.20]	<0.001	42.3
Chronic back pain	Anxiety	1.71 [1.36,2.14]	<0.001	1.61 [1.28,2.03]	<0.001	1.06 [1.03,1.09]	<0.001	10.6
	Cognition	1.74 [1.38,2.18]	<0.001	1.48 [1.18,1.86]	0.001	1.17 [1.12,1.23]	<0.001	28.7
	Depression	1.71 [1.37,2.14]	<0.001	1.65 [1.32,2.07]	<0.001	1.03 [1.01,1.06]	0.003	6.2
	Sleep/energy	1.70 [1.36,2.13]	<0.001	1.50 [1.19,1.89]	0.001	1.13 [1.08,1.18]	<0.001	23.6
Chronic lung disease	Anxiety	1.48 [1.18,1.85]	0.001	1.39 [1.11,1.74]	0.005	1.07 [1.04,1.10]	<0.001	16.4
	Cognition	1.48 [1.19,1.84]	<0.001	1.33 [1.07,1.66]	0.011	1.11 [1.08,1.15]	<0.001	27.6
	Depression	1.48 [1.19,1.85]	0.001	1.43 [1.15,1.79]	0.001	1.03 [1.01,1.05]	0.002	8.2
	Sleep/energy	1.49 [1.19,1.85]	<0.001	1.32 [1.06,1.65]	0.013	1.13 [1.08,1.17]	<0.001	29.9
Hearing problems	Anxiety	1.58 [1.24,2.03]	<0.001	1.54 [1.21,1.97]	0.001	1.03 [1.00,1.05]	0.036	6.0
	Cognition	1.60 [1.24,2.05]	<0.001	1.36 [1.06,1.75]	0.015	1.17 [1.12,1.22]	<0.001	33.4
	Depression	1.57 [1.22,2.02]	0.001	1.57 [1.21,2.02]	0.001	1.00 [0.99,1.02]	0.842	NA
	Sleep/energy	1.59 [1.24,2.05]	<0.001	1.50 [1.17,1.94]	0.002	1.06 [1.03,1.09]	<0.001	12.2
Stroke	Anxiety	2.02 [1.52,2.69]	<0.001	1.97 [1.48,2.61]	<0.001	1.03 [1.00,1.06]	0.054	NA
	Cognition	2.03 [1.56,2.66]	<0.001	1.75 [1.33,2.29]	<0.001	1.16 [1.11,1.23]	<0.001	21.5
	Depression	2.03 [1.54,2.66]	<0.001	2.00 [1.52,2.63]	<0.001	1.01 [1.00,1.03]	0.112	NA
	Sleep/energy	2.03 [1.56,2.64]	<0.001	1.90 [1.46,2.48]	<0.001	1.07 [1.03,1.11]	<0.001	9.3
Visual impairment	Anxiety	2.64 [1.75,3.99]	<0.001	2.10 [1.38,3.21]	0.001	1.26 [1.13,1.39]	<0.001	23.5
	Cognition	2.66 [1.73,4.10]	<0.001	1.97 [1.27,3.06]	0.002	1.35 [1.23,1.48]	<0.001	30.7
	Depression	2.60 [1.73,3.91]	<0.001	2.43 [1.62,3.65]	<0.001	1.07 [1.02,1.12]	0.009	6.9
	Sleep/energy	2.64 [1.75,3.99]	<0.001	2.29 [1.51,3.47]	<0.001	1.15 [1.08,1.23]	<0.001	14.6
Multimorbidity^a^	Anxiety	1.41 [1.20,1.67]	<0.001	1.36 [1.15,1.60]	<0.001	1.04 [1.02,1.06]	<0.001	11.5
	Cognition	1.40 [1.19,1.65]	<0.001	1.24 [1.05,1.47]	0.012	1.12 [1.09,1.16]	<0.001	35.0
	Depression	1.41 [1.19,1.66]	<0.001	1.37 [1.16,1.62]	<0.001	1.03 [1.01,1.04]	0.001	7.5
	Sleep/energy	1.40 [1.19,1.66]	<0.001	1.23 [1.05,1.45]	0.012	1.14 [1.09,1.19]	<0.001	38.1

*Abbreviation*: *OR* Odds ratio, *CI* Confidence interval

Highly sedentary behavior referred to being sedentary for ≥8 h/day

Models are adjusted for sex, age, education, wealth, setting, unemployment, living arrangement, and country. For individual chronic conditions, the models were also adjusted for the presence of other chronic conditions

The mediated percentage was only calculated in the presence of a significant indirect effect (*P* < 0.05)

^a^Multimorbidity was defined as ≥2 chronic conditions

**Table 4:** Disability, mobility, and pain/discomfort as mediators in the association between chronic conditions (multimorbidity) and highly sedentary behavior among adults aged ≥50 years

		Total effect		Direct effect		Indirect effect		
Exposure	Mediator	OR [95% CI]	*P*-value	OR [95% CI]	*P*-value	OR [95% CI]	*P*-value	% Mediated
Arthritis	Disability	1.23 [1.04,1.46]	0.017	1.04 [0.87,1.24]	0.670	1.18 [1.14,1.23]	<0.001	81.6
	Mobility	1.23 [1.04,1.45]	0.015	1.02 [0.86,1.22]	0.780	1.20 [1.15,1.25]	<0.001	88.1
	Pain/discomfort	1.23 [1.04,1.46]	0.016	1.03 [0.87,1.22]	0.732	1.19 [1.14,1.25]	<0.001	85.6
Asthma	Disability	1.41 [1.07,1.85]	0.014	1.07 [0.81,1.41]	0.638	1.32 [1.24,1.40]	<0.001	80.6
	Mobility	1.41 [1.08,1.84]	0.011	1.12 [0.86,1.45]	0.413	1.27 [1.20,1.33]	<0.001	68.4
	Pain/discomfort	1.41 [1.08,1.84]	0.011	1.21 [0.93,1.58]	0.158	1.17 [1.11,1.22]	<0.001	44.5
Chronic back pain	Disability	1.71 [1.35,2.17]	<0.001	1.28 [1.02,1.60]	0.035	1.34 [1.26,1.43]	<0.001	54.7
	Mobility	1.75 [1.38,2.22]	<0.001	1.36 [1.08,1.72]	0.009	1.29 [1.22,1.36]	<0.001	44.9
	Pain/discomfort	1.73 [1.37,2.17]	<0.001	1.36 [1.09,1.69]	0.007	1.27 [1.19,1.36]	<0.001	43.8
Chronic lung disease	Disability	1.49 [1.19,1.86]	<0.001	1.16 [0.93,1.45]	0.191	1.28 [1.22,1.35]	<0.001	62.5
	Mobility	1.50 [1.21,1.87]	<0.001	1.19 [0.96,1.46]	0.110	1.27 [1.21,1.33]	<0.001	58.1
	Pain/discomfort	1.49 [1.20,1.85]	<0.001	1.27 [1.02,1.58]	0.032	1.18 [1.12,1.23]	<0.001	40.4
Hearing problems	Disability	1.58 [1.21,2.05]	0.001	1.19 [0.91,1.55]	0.194	1.32 [1.24,1.42]	<0.001	61.4
	Mobility	1.60 [1.23,2.07]	<0.001	1.35 [1.04,1.75]	0.024	1.18 [1.13,1.24]	<0.001	35.8
	Pain/discomfort	1.59 [1.23,2.05]	<0.001	1.46 [1.13,1.87]	0.003	1.09 [1.05,1.13]	<0.001	18.6
Stroke	Disability	2.01 [1.52,2.67]	<0.001	1.40 [1.04,1.87]	0.026	1.44 [1.32,1.58]	<0.001	52.3
	Mobility	2.02 [1.54,2.65]	<0.001	1.48 [1.12,1.98]	0.007	1.36 [1.27,1.46]	<0.001	43.8
	Pain/discomfort	2.04 [1.57,2.66]	<0.001	1.83 [1.41,2.39]	<0.001	1.11 [1.07,1.17]	<0.001	15.2
Visual impairment	Disability	2.79 [1.85,4.22]	<0.001	1.35 [0.88,2.05]	0.165	2.08 [1.77,2.43]	<0.001	71.1
	Mobility	2.76 [1.80,4.25]	<0.001	1.79 [1.16,2.74]	0.008	1.55 [1.40,1.71]	<0.001	43.0
	Pain/discomfort	2.68 [1.77,4.06]	<0.001	2.09 [1.38,3.17]	0.001	1.28 [1.19,1.39]	<0.001	25.4
Multimorbidity^a^	Disability	1.36 [1.15,1.61]	<0.001	1.03 [0.87,1.23]	0.705	1.32 [1.25,1.39]	<0.001	89.1
	Mobility	1.39 [1.18,1.64]	<0.001	1.05 [0.89,1.24]	0.570	1.32 [1.26,1.40]	<0.001	85.1
	Pain/discomfort	1.40 [1.18,1.66]	<0.001	1.14 [0.97,1.35]	0.119	1.22 [1.16,1.30]	<0.001	60.2

*Abbreviation*: *OR* Odds ratio, *CI* Confidence interval

Highly sedentary behavior referred to being sedentary for ≥8 h/day

Models are adjusted for sex, age, education, wealth, setting, unemployment, living arrangement, and country. For individual chronic conditions, the models were also adjusted for the presence of other chronic conditions

The mediated percentage was only calculated in the presence of a significant indirect effect (*P* < 0.05)

^a^Multimorbidity was defined as ≥2 chronic conditions

For the individual chronic conditions, anxiety did not explain the association of high SB with arthritis and stroke while for other conditions it explained from 6.0% (hearing problems) to 23.5% (visual impairment) of the association. Cognitive problems explained the relationship from 21.5% (stroke) to 33.4% (hearing problems). Depression was not an influential factor in the association of high SB with hearing problems and stroke, while it explained between 6.2% (chronic back pain) to 13.2% (asthma) of the association for the other conditions. Sleep/energy problems explained 9.3% (stroke) to 49.1% (arthritis) of the relationship. Disability explained more than 50% of the association for all chronic conditions with particularly high mediated percentages observed for arthritis (81.6%) and asthma (80.6%). Mobility difficulties also explained more than 35% of the association for all chronic conditions with particularly high mediated percentages observed for arthritis (88.1%). Pain was a major influential factor for the association between SB and arthritis (85.6%) and explained between 15.2% (stroke) to 44.5% (asthma) of the association with other conditions.

As for the SB - physical multimorbidity relationship, the most important influential factor was disability (89.1%), followed by mobility (85.1%), pain/discomfort (60.2%), sleep/energy (38.1%), cognition (35.0%), anxiety (11.5%), and depression (7.5%).

## Discussion

### General findings

To the best of our knowledge, the current study is the first large-scale (*n* = 34,129), multinational (6 LMICs) analysis investigating associations of SB with chronic conditions and physical multimorbidity. The current data confirm the findings from a previous study conducted in a high-income country showing that SB is associated with a higher risk for physical multimorbidity [20]. We found that most chronic conditions were associated with a high SB status (i.e. eight or more hours per day) in the overall sample, although this relationship was most notable among the oldest population (i.e. 65 years or older). Our mediation analysis showed that disability and mobility difficulties were important factors for most of the chronic conditions studied, while pain was a central factor for arthritis. As for physical multimorbidity, disability, mobility difficulties and pain were also important factors mediating high SB. Sleep/energy and cognitive problems explained the relation between SB and each chronic condition to a lesser extent while findings for anxiety, and depression were mixed. The factors identified in our study, which may explain the relationship between chronic conditions and physical multimorbidity with SB, should however be confirmed in prospective studies.

In the overall sample, those with arthritis (OR = 1.22) and chronic back pain (OR = 1.70) were more likely to be sedentary for eight or more hours per day. These relationships appear to be mainly explained by disability, mobility difficulties, and pain while cognitive problems, depressive feelings, and sleep problems may also play a role. In both conditions, pain might cause mobility and sleep problems and ultimately feelings of depression, which in turn can all result in people being more sedentary. Previous literature in Western populations has demonstrated that pain can result in mobility limitations, higher levels of SB [47], and depression [48]. Clearly, such an interplay of pain, mobility limitations and depression among people with physical multimorbidity could play an even more pronounced role. Next to this, some SBs such as TV viewing, have been linked previously to poorer cognitive function in middle-aged to older adults [49], although this is largely drawn from high-income populations. The reason why adults with cognitive problems are more likely to be sedentary is largely unknown, but clearly this warrants further exploration. One possible explanation is that cognitive problems, such as impairments in executive functioning can result in difficulties in planning complex behaviors and consequently there might be an increased risk of falls [50]. Falls are on its turn associated with a fear of falling again and avoidance of activities due to this fear, which is also associated with grey matter volume loss [51, 52]. In the case of arthritis, cognitive problems may be linked to SB via comorbid cardiovascular risk factors or systemic inflammatory processes [53, 54]. Sedentary time and the number of sedentary bouts per day (i.e. ≥20 min) are associated with an increased 10-year cardiovascular disease (CVD) risk in people with arthritis, independent of engagement in moderate to vigorous physical activity [55].

Our data also suggest that people with asthma were more sedentary (OR = 1.39). It is known that many individuals with asthma avoid physical activities for fear of exacerbating symptoms or triggering physical activity-induced bronchoconstriction [56]. Studies have shown that replacing SB with light intensity physical activity can however improve asthma control and asthma-related quality of life [56], and is a possible protective factor against asthma development [57]. As people with asthma are at an increased risk for mobility problems and musculoskeletal pain [58], interventions focusing on reducing this pain, discomfort and mobility problems are probably essential as well to reduce the time spent sedentary. Not only asthma, but also other chronic lung diseases such as chronic obstructive pulmonary disease (COPD) may induce mobility difficulties, pain, or disability. These factors may predispose an individual to be more socially isolated due to their restrictions in ability to conduct activities of daily living or stigmatized as respiratory symptoms are often associated with tuberculosis in LMICs [59].

For over a decade, targeting increases in moderate to vigorous physical activity has been the emphasis of a large number of exercise training and behavior change interventions in people with COPD [60], but with limited success [61]. The lower levels of physical activity coupled with the often fragile physical and psychological health among patients with COPD may make reducing SB a more suitable conduit for behavior change [62, 63]. In our study, people with COPD had 1.48 times higher odds for being sedentary eight or more hours. Recent evidence showed as well that SB is an independent predictor of mortality in subjects with COPD, even adjusting for moderate-to-vigorous physical activity and a number of other variables. Mortality was four times higher in subjects with COPD who spend ≥8.5 h/d in activities requiring <1.5 MET [64]. Therefore, there is an urgent need to test interventions to reduce SB including the provision of information about the health consequences of SB [65].

Stroke was associated with a 2.0 times higher odds for high SB in our study. A previous qualitative study in Canadian stroke survivors [66] demonstrated that there is also limited awareness of health risks of SB among stroke survivors.

Finally, we also found significant associations between highly sedentary status and hearing problems and visual impairments. Hearing problems [67] and visual impairments [68] should therefore be considered as important risk conditions for SB in LMICs. Stigma and discrimination associated with these chronic conditions and a lack of social support may complicate daily life participation in these populations.

### Practical implications and future research

Our analyses provide further evidence for the need to develop strategies to deal with a sedentary lifestyle in LMICs, and offer important hypotheses for testing in future prospective cohort studies. Our data add to the need to increase awareness among care providers at all levels of care in LMICs of the risks of SB of their patients. Given the evidence in the general population of the benefits of reducing SB, we propose that it is important to test the efficacy and effectiveness of a dual strategy in low resource settings by developing both a smaller group of master trainers/supervisors (e.g. exercise physiologists and physiotherapists) and researchers and a larger group of practitioners (e.g. nurses) who are responsible for minimizing SB in high risk groups. This method has been successfully employed for cognitive behavioral therapy in trials in LMICs [69, 70]. A stepped-care approach, where patients start with self-management in order to reduce their SB, may be a feasible strategy in LMIC settings. Such self-management strategies could, for example, include regular interruptions in sedentary time, such as standing or walking frequently throughout waking hours, during television commercial breaks or while using the phone. Then, if patients do not achieve significant reductions, they could continue with a manualized approach under the supervision of a non-specialist worker (e.g. nurses, occupational therapists). Patients would only be referred to a specialist supervisor (e.g. exercise physiologists and physiotherapists) if no significant reductions occur, for example due to pain, sleep and cognitive problems or mobility problems or associated anxiety and depression. It is known from physical activity research that inclusion of exercise physiologists or physiotherapists reduces dropout rates from lifestyle interventions and consequently improves outcomes [71]. Careful consideration of which strategies would be most efficacious, and evaluation of this stepped-care approach, is essential. The current available evidence is, however, mainly based on evidence from high-income countries and showed that lifestyle interventions focusing on light physical activity participation reduced SB by 24 min/day (95%CI = 8 to 41 min/day, *n* = 3981) while in interventions focusing on reducing time spent sedentary only, SB was reduced by 42 min/day (95% CI = 5 to 79 min/day, *n* = 62) [72].

Although application of self-monitoring devices and alarms are not easy implementable in low resource settings, a recent International mobile-health Intervention on physical activity, sitting, and weight in adult employees using light-weight, low-cost, non-interactive pedometers as a self-monitoring and motivational tool demonstrated reduction in sedentary time. In a 100-day program, participants were encouraged to increase incidental activity such as using stairs and avoidance of sitting. After 100 days, the time spent sedentary reduced in low-income countries by 0.73 h per day (95% CI = −0.75 to −0.70 h) and by 0.59 h per day 95% CI = −1.08 to −0.10 h) in middle-income countries [73].

Efficacy trials of interventions focusing on SB among people with chronic conditions in different cultural settings across LMICs are however urgently needed. If the efficacy and effectiveness of these SB interventions are well established in better equipped scientific settings with trained research staff, the final step will be to fund interventions and initiatives to translate research findings into “real-world” settings while exploring its cost-effectiveness. In order to justify the inclusion of programs focusing on SB reductions as a routine component in the treatment of chronic diseases and multimorbidity in LMICs, cost-benefit analyses should be conducted in order to quantify the financial implications of diverting resources or investing funds into such initiatives. Therefore, next to intervention studies exploring the efficacy of these programs, effectiveness research capable of driving practice change, along with policy-level research, is urgently required. Ministries of health and education will play a critical role in this governance and policy development step. If research shows that reducing SB is efficacious and effective in the prevention and management of chronic diseases and multimorbidity in middle-aged to older adults in LMICs, interventions should be mainstreamed in existing health systems at all levels of care. Finally, since visual impairments and hearing problems are associated with higher levels of SB, future research could explore whether programs providing hearing aids and glasses might assist those in need to become more physically active.

### Limitations and strengths

The current findings should be interpreted in light of some limitations. First, the study is cross-sectional, and cause and effect cannot be deduced. Therefore, it remains unclear whether SB was caused by chronic conditions or vice versa. For example, SB is known to be a risk factor for cardiovascular diseases, while pain caused by arthritis may cause people to be more sedentary. Second, whilst we included all physical health conditions which were assessed within the SAGE, other physical conditions such as tuberculosis, cancer and HIV may have been present and not identified in the study. Therefore, the prevalence of physical multimorbidity is likely to be an underestimation and it is possible that the association between multimorbidity and SB could have differed if data on more chronic physical conditions were available. Third, since the information on chronic conditions and SB was based on self-report, reporting biases may exist. Future research should utilize objective measures of SB. Accelerometers-inclinometers are available that allow for valid and reliable SB behavior. Fourth, by separating the sample into dichotomous SB categories, we were not able to examine how different quantities of SB may affect morbidity. Finally, the present study did not include institutionalized people, which may limit generalizability at a national level. Nonetheless, the strengths of the study include the multi-national scope focused on LMICs, countries which are not investigated yet in the prior research literature. Additionally, we investigated numerous potentially modifiable factors that can be targeted for future interventions aiming to reduce SB.

## Conclusions

The current study demonstrates that middle-aged to older adults with chronic conditions and physical multimorbidity are significantly more sedentary. Future longitudinal research is required to confirm the directionality of our results and explore influential factors. In addition, research on the efficacy and effectiveness of reducing SB in the management of chronic diseases and physical multimorbidity in LMICs should be a priority for funding bodies. Clinicians should consider in particular mobility problems, disability and pain as potential barriers but also other mental health issues such as sleep problems and poor cognition.

## Additional file

Additional file 1: Table S1. Questions used to assess self-reported diagnosis. **Table S2.** Questions and answer options used for symptoms-based diagnosis. **Table S3.** Questions used to assess health status. **Table S4.** Questions and answer options used for endorsement of DSM-IV depression. (DOCX 15 kb)

## Abbreviations

CI: Confidence interval
HICs: High-income countries
LMICs: Low- and middle-income countries
OR: Odds ratio
SAGE: Study on Global Ageing and Adult Health
SB: Sedentary behavior
