# Supplementary material for: Physical chronic conditions, multimorbidity and sedentary behavior amongst middle-aged and older adults in six low- and middle-income countries
Source: Int J Behav Nutr Phys Act. 2017 Oct 27;14:147. doi: 10.1186/s12966-017-0602-z (PMC5658996; doi:10.1186/s12966-017-0602-z)
Supplement: Additional file 1: Table S1. — Questions used to assess self-reported diagnosis. Table S2. Questions and answer options used for symptoms-based diagnosis. Table S3. Questions used to assess health status. Table S4. Questions and answer options used for endorsement of DSM-IV depression. (DOCX 15 kb) [file 12966_2017_602_MOESM1_ESM.docx]

**Additional file 1**

**Table S1** Questions used to assess self-reported diagnosis

| Condition | Question |
| --- | --- |
| Angina | Have you ever been diagnosed with angina or angina pectoris (a heart disease)? |
| Arthritis | Have you ever been diagnosed with/told you have arthritis (a disease of the joints, or by other names rheumatism or osteoarthritis)? |
| Asthma | Have you ever been diagnosed with asthma (an allergic respiratory disease)? |
| Chronic lung disease | Have you ever been diagnosed with chronic lung disease (emphysema, bronchitis, COPD)? |
| Diabetes | Have you ever been diagnosed with diabetes (high blood sugar)? (not including diabetes associated with a pregnancy) |
| Hypertension | Have you ever been diagnosed with high blood pressure (hypertension)? |
| Stroke | Have you ever been told by a health professional that you have had a stroke? |

**Table S2** Questions and answer options used for symptoms-based diagnosis

| **Condition** | **Symptom-based algorithm** |
| --- | --- |
| Angina | Rose questionnaire |
| Arthritis | Affirmative answers to all four of the following:  1. During the last 12 months, have you experienced pain, aching, stiffness or swelling in or around the joints (e.g., in arms, hands, legs or feet) which were not related to an injury and lasted for more than a month?  2. During the last 12 months, have you experienced stiffness in the joint in the morning after getting up from bed, or after a long rest of the joint without movement?  3. Did this stiffness last for less than 30 minutes?  4. Did this stiffness go away after exercise or movement in the joint? |
| Asthma | 1. During the last 12 months, have you experienced attacks of wheezing or whistling breathing? (Yes)  **AND**  2. “Yes” to at least one of the following (past 12 months):  (a) Have you experienced an attack of wheezing that came on after you stopped exercising or some other physical activity?  (b) Have you had a feeling of tightness in your chest?  (c) Have you woken up with a feeling of tightness in your chest in the morning or any other time?  (d) Have you had an attack of shortness of breath that came on without an obvious cause when you were not exercising or doing some physical activity? |
| Chronic lung disease | 1. During the last 12 months, have you experienced any shortness of breath at rest (while awake)?  (Yes)  **OR**  2. “Yes” to both of the following (past 12 months):  (a) Have you experienced any coughing or wheezing for 10 minutes or more at a time?  (b) Have you experienced any coughing up of sputum or phlegm on most days of the month for at least 3 months? |

**Table S3** Questions used to assess health status

| **Mobility** | (1) Overall in the last 30 days, how much difficulty did you have with moving around? |
| --- | --- |
|  | (2) Overall in the last 30 days, how much difficulty did you have in vigorous activities, such as running 3 km (or equivalent) or cycling? |
| **Pain and discomfort** | (1) Overall in the last 30 days, how much of bodily aches or pains did you have?  (2) Overall in the last 30 days, how much bodily discomfort did you have? |
| **Cognition** | (1) Overall in the last 30 days, how much difficulty did you have with concentrating or remembering things? |
|  | (2) Overall in the last 30 days, how much difficulty did you have in learning a new task (for example, learning how to get to a new place, learning a new game, learning a new recipe etc.)? |
| **Sleep and energy** | (1) Overall in the last 30 days, how much of a problem did you have with sleeping, such as falling asleep, waking up frequently during the night or waking up too early in the morning? |
|  | (2) Overall in the last 30 days, how much of a problem did you have due to not feeling rested and refreshed during the day (e.g. feeling tired, not having energy)? |

**Table S4** Questions and answer options used for endorsement of DSM-IV depression

| 1. At least one of the two following symptoms in the last 12 months: |
| --- |
| (a) A period, lasting several days, of feeling sad, empty or depressed. |
| (b) A period lasting several days with a loss of interest in most things the participant usually enjoys such as personal relationships, work or hobbies/recreation. |
| **AND** |
| 2. The period of sadness/loss of interest/low energy lasted for more than two weeks and was most of the day and nearly every day. |
| **AND** |
| 3. Five or more of the following symptoms: |
| (a) Loss of appetite |
| (b) Insomnia (problems falling asleep or waking up too early) |
| (c) Decreased energy or tiredness all the time |
| (d) Slowing down in moving around or restless/jittery. |
| (e) Negative feelings/loss of confidence or frequent feelings of hopelessness. |
| (f) Slowed thinking or difficulties concentrating (e.g., listening to others, working, watching TV, listening to the radio). |
| (g) Thoughts of death, wishes of own death or suicide attempt. |
| (h) Feelings of sadness, emptiness or depression lasting several days. |
| (i) Anhedonia: loss of interest in things the participant usually enjoys. |
